# Enhancing CO_2_ Mineralization and Mechanical Performance of Fly Ash-Based Alkali-Activated Mortar Using Calcined Seashell Ash

**DOI:** 10.3390/ma19204325

**Published:** 2026-10-14

**Authors:** Darrakorn Intarabut, Piti Sukontasukkul, Suthatip Sinyoung, Manote Sappakittipakorn, Tanakorn Phoo-ngernkham, Worathep Sae-Long, Avirut Puttiwongrak, Suchart Limkatanyu

**Affiliations:** 1 Department of Civil Engineering, Rajamangala University of Technology Phra Nakhon, Bangkok 10800, Thailand; darrakorn.i@rmutp.ac.th; 2 Construction and Building Materials Research Center, Department of Civil Engineering, Faculty of Engineering, King Mongkut’s University of Technology North Bangkok, Bangkok 10800, Thailand; manote.s@eng.kmutnb.ac.th; 3 Department of Civil and Environmental Engineering, Faculty of Engineering, Prince of Songkla University, Songkhla 90110, Thailand; suthatip.s@psu.ac.th (S.S.); suchart.l@psu.ac.th (S.L.); 4 Sustainable Construction Material Technology Research Unit, Department of Civil Engineering, Faculty of Engineering and Technology, Rajamangala University of Technology Isan, Nakhon Ratchasima 30000, Thailand; tanakorn.ph@rmuti.ac.th; 5 Civil Engineering Program, School of Engineering, University of Phayao, Phayao 56000, Thailand; worathep.sa@up.ac.th; 6 Geotechnical and Earth Resources Engineering, Faculty of Civil and Environmental Engineering, Asian Institute of Technology, Pathumthani 12120, Thailand; avirut@ait.ac.th

**Keywords:** calcined seashell ash, alkali-activated mortar, CO_2_ mineralization, carbonation, waste valorization

## Abstract

This study investigated the use of calcined seashell ash (CSA) as a Ca-rich modifier to enhance the mechanical performance and CO_2_ mineralization of high-calcium fly ash-based alkali-activated mortar. CSA was incorporated at 0–20% replacement levels, while liquid-to-binder (L/B) ratios of 0.35, 0.40, and 0.45 were investigated. Fresh and mechanical properties were evaluated through spread flow, initial setting time, compressive strength, and flexural strength tests. Mineral carbonation was assessed using accelerated carbonation depth, X-ray diffraction (XRD), and thermogravimetric/derivative thermogravimetric (TG/DTG) analyses. Increasing CSA content generally reduced flowability and shortened initial setting time, while 15% CSA produced the highest mean 28-day mechanical strengths. At this level, the 28-day compressive strength increased by 11.5–38.8%, depending on the L/B ratio, while the 28-day flexural strength increased by approximately 102–107% relative to the corresponding mixtures without CSA. Carbonation behavior was strongly influenced by the L/B ratio. At 20% CSA replacement, the carbonation-depth enhancement factor (CEF_x_) relative to the corresponding mixture without CSA increased from only 1.2% at L/B = 0.35 to 13.2% and 16.6% at L/B = 0.40 and 0.45, respectively. XRD revealed substantially increased calcite-related diffraction features following accelerated carbonation, particularly in the CSA-containing mixtures, while TG/DTG showed a stronger carbonation-associated thermal response for the mixture containing 20% CSA at L/B = 0.45 than for the corresponding mixture without CSA. Together with the carbonation-depth results, these complementary observations consistently indicate that CSA enhanced CO_2_ mineralization through increased carbonate formation under accelerated CO_2_ exposure. However, the absolute amount of chemically bound CO_2_ was not directly quantified in the present study. Overall, CSA can function as both a partial fly ash replacement and a Ca-rich modifier, with its mineral carbonation response governed by the balance between the availability of Ca-bearing phases and CO_2_ accessibility within the hardened matrix.

## 1. Introduction

Human-caused greenhouse gas emissions have intensified global climate change, with the global surface temperature reaching approximately 1.1 °C above the 1850–1900 level during 2011–2020 [1], highlighting the urgent need for effective strategies to reduce carbon dioxide (CO_2_) emissions across major industrial sectors. Among the major industrial sectors, the buildings and construction sector has an intensely large environmental footprint because of its extensive consumption of energy and construction raw materials. Recent estimates indicate that the sector accounts for approximately 37% of global CO_2_ emissions and nearly 50% of global material extraction [2]. The production of cement and concrete is of particular concern because of the enormous production volume and the CO_2_ emissions associated with cement manufacture from both fuel combustion and the calcination of carbonate raw materials. Consequently, moving toward reducing the carbon footprint of cementitious construction materials has become one of the important global efforts toward a low-carbon built environment.

During the construction phase, large amounts of CO_2_ are emitted directly from cement and concrete production, as well as from fossil fuel consumption by construction machinery and transportation. Despite the substantial CO_2_ emissions during the construction phase, cementitious materials can partially reabsorb CO_2_ from the atmosphere through carbonation during their service life and after demolition. Carbonation occurs when CO_2_ penetrates the pore structure of a cementitious matrix, dissolves in the pore solution, and reacts with calcium-bearing phases to form carbonate products, mostly calcium carbonate (CaCO_3_) [3,4,5,6]. In Portland cement-based materials, calcium hydroxide (Ca(OH)_2_) and calcium–silicate–hydrate (C–S–H) phases provide important sources of calcium for these reactions [3,4,6]. Although carbonation has traditionally been regarded as a durability concern in reinforced concrete because the associated reduction in pore-solution alkalinity can promote reinforcement corrosion, the same chemical process provides a mechanism for permanent mineral sequestration of atmospheric CO_2_ [3,4]. Andrade and Sanjuán [6] demonstrated measurable CO_2_ uptake in Portland and blended cement pastes and indicated that the CO_2_ uptake capacity depends on the water-to-cement (w/c) ratio and exposure conditions, including indoor or outdoor environments and relative humidity. At the global scale, Xi et al. [5] estimated that carbonation of cement materials between 1930 and 2013 sequestered approximately 4.5 GtC, offsetting about 43% of the process CO_2_ emissions from cement production over the same period. Their findings indicated that carbonation of cementitious materials represents a substantial carbon sink that has long been overlooked in conventional emissions inventories. This inherent capacity has therefore attracted increasing interest as a complementary pathway for reducing the net carbon footprint of cementitious construction materials.

Alkali-activated binders (AABs), including geopolymer systems, have received considerable attention as sustainable alternatives to ordinary Portland cement because they can utilize aluminosilicate-rich industrial by-products, such as fly ash and ground granulated blast-furnace slag, while reducing the consumption of Portland cement and associated CO_2_ emissions [7,8,9]. In fly ash-based AABs, the reaction of aluminosilicate precursors with alkaline activators leads predominantly to the formation of sodium aluminosilicate hydrate (N–A–S–H) gel, whereas calcium-rich precursor systems may additionally form calcium–aluminosilicate–hydrate (C–A–S–H) or hybrid gel phases [9,10,11]. From the perspective of CO_2_ mineralization, however, low-calcium fly ash-based AABs contain substantially less readily available calcium than Portland cement or slag-rich systems. Since stable mineral fixation of CO_2_ in cementitious materials is strongly associated with the carbonation of calcium-bearing phases, the limited availability of reactive calcium may restrict the potential CO_2_ uptake of low-calcium fly ash-based AABs [11,12,13]. Introducing an appropriate external calcium source may therefore provide a means of enhancing their mineral carbonation capacity while retaining the environmental advantages associated with alkali-activated binder technology.

The incorporation of calcium-rich constituents has been widely investigated as a means of modifying the reaction chemistry and performance of alkali-activated and geopolymer binders. The presence of calcium can promote the formation of Ca-containing reaction products alongside the aluminosilicate gel network, thereby influencing setting behavior, microstructure, and mechanical properties [14,15,16]. From the perspective of CO_2_ utilization, the availability of reactive calcium is particularly important because Ca-bearing phases can react with CO_2_ during carbonation to form thermodynamically stable calcium carbonate (CaCO_3_), providing a mechanism for permanent mineral fixation of CO_2_. Hossain and Akhtar [17] highlighted that the CO_2_ capture and storage potential of geopolymers is governed by several interacting factors, including precursor chemistry, reaction products, pore characteristics, and exposure conditions. Therefore, increasing the availability of reactive calcium may provide a potential route for enhancing CO_2_ mineralization in alkali-activated binders. However, the effectiveness of this approach depends not only on the total calcium content but also on its chemical form and reactivity, the resulting phase assemblage and pore structure, and the conditions under which carbonation occurs. Selection of an appropriate calcium-rich constituent is therefore important for enhancing CO_2_ uptake while maintaining desirable engineering performance.

Among potential calcium-rich constituents, waste seashells offer an attractive alternative because they are generated in large quantities by seafood consumption and processing and consist predominantly of calcium carbonate (CaCO_3_). Thermal treatment of seashells can promote the decomposition of CaCO_3_ and produce Ca-bearing phases with increased reactivity, providing a potential reactive calcium source while simultaneously valorizing a biological waste stream. Seesanong et al. [18] demonstrated the production of high-purity CaO from waste oyster shells through controlled thermal decomposition, confirming their potential as a low-cost and waste-derived calcium resource. The feasibility of incorporating calcined seashells into alkali-activated binders has also been demonstrated. Djobo et al. [15] reported that partial replacement of volcanic ash with calcined oyster shell influenced the setting, microstructure, and mechanical properties of volcanic ash-based geopolymers, while Yankwa Djobo et al. [16] further demonstrated that oyster-shell-derived calcium significantly affected the gel composition and strength development of alkali-activated oyster shell–volcanic ash systems.

These studies establish the potential of calcined seashells as Ca-rich constituents in alkali-activated materials; however, their primary emphasis has been on reaction mechanisms and engineering properties rather than their deliberate use for enhancing CO_2_ mineralization. In particular, the contribution of calcined seashell-derived calcium to CO_2_ uptake in an inherently high-calcium fly ash-based alkali-activated binder remains insufficiently understood. Investigating such a combined calcium system may provide new insights into how intrinsic calcium from fly ash and additional waste-derived calcium from calcined seashells influence carbonate formation, CO_2_ uptake, and engineering performance.

Accordingly, this study investigates the use of calcined seashell ash (CSA) as a Ca-rich reactive modifier in high-calcium fly ash-based alkali-activated mortar. The effects of CSA incorporation and liquid-to-binder ratio on fresh properties, setting behavior, compressive and flexural strengths, and accelerated carbonation depth are evaluated. X-ray diffraction (XRD) and thermogravimetric/derivative thermogravimetric (TG/DTG) analyses are further employed to examine carbonate formation and elucidate the Ca-mediated mineral carbonation response. The study aims to determine whether waste-derived CSA can enhance CO_2_ mineralization while maintaining desirable engineering performance, thereby providing a combined pathway for carbon management and seashell-waste valorization.

## 2. Materials and Experimental Program

### 2.1. Raw Materials

In this study, high-calcium fly ash (FA) obtained from the Mae Moh Power Plant (Mae Moh, Lampang, Thailand), and calcined seashell ash (CSA) were used as the primary binder materials for producing alkali-activated mortar. The FA had a specific gravity of 2.61 and an average particle size of 14.6 µm.

Discarded seashells collected from the marine food industry were used as the source material for producing CSA. The shells were first washed thoroughly with water to remove adhering impurities and organic residues, and then air-dried before calcination. The cleaned shells were calcined in an electric furnace at 800 °C for 2 h to promote the thermal decomposition of CaCO_3_ and increase the availability of Ca-bearing phases. After calcination, the material was allowed to cool naturally to room temperature inside the furnace for approximately 24 h, followed by grinding into a fine powder. The chemical compositions determined by X-ray fluorescence (XRF) are presented in Table 1. It should be noted that the CaO value obtained by XRF represents the calcium content expressed on an oxide-equivalent basis and does not directly identify the mineralogical form or reactivity of the Ca-bearing phases present in CSA.

The fly ash-based alkali-activated mortar was activated using a combination of sodium hydroxide (NaOH) solution and sodium silicate (Na_2_SiO_3_) solution. Sodium hydroxide pellets were dissolved in water to prepare a 12 M NaOH solution. The commercial sodium silicate solution contained 34.2% SiO_2_, 16.3% Na_2_O, and 49.5% H_2_O by mass. River sand with a specific gravity of 2.51 and a fineness modulus of 3.73 was used as the fine aggregate. The mass ratio of sodium silicate solution to sodium hydroxide solution (S/H) was maintained at 1.0 throughout the study.

### 2.2. Mixture Proportions

A total of twelve mortar mixtures were prepared using a 4 × 3 factorial experimental design to investigate the effects of calcined seashell ash (CSA) replacement and liquid-to-binder (L/B) ratio on the properties of fly ash-based alkali-activated mortar. Calcined seashell ash (CSA) was used as a partial replacement for fly ash (FA) at replacement levels of 0%, 10%, 15%, and 20% by mass of the total binder. The total binder content was maintained constant at 772 kg/m^3^ for all mixtures.

To evaluate the influence of workability and reaction kinetics, three liquid-to-binder (L/B) ratios of 0.35, 0.40, and 0.45 were investigated. The L/B ratio was defined as the mass ratio of the total alkaline activator solution (NaOH solution + Na_2_SiO_3_ solution) to the total binder (FA + CSA). The mass ratio of sodium silicate solution to sodium hydroxide solution (S/H) was maintained at 1.0 for all mixtures. The fine aggregate-to-binder ratio was fixed at 1.5 throughout the study. The mixture identification consisted of two components. The first number represents the percentage of CSA replacing FA by mass, while the second number denotes the corresponding liquid-to-binder ratio. For example, G10/0.35 represents a mixture containing 10% CSA replacement with an L/B ratio of 0.35. The detailed mixture proportions are presented in Table 2.

The actual W/B ratio was calculated from the water contained in both the 12 M NaOH solution and the sodium silicate solution relative to the total binder mass. The activator Na_2_O/B ratio was calculated from the Na_2_O equivalents supplied by the NaOH and sodium silicate solutions relative to the total binder mass; Na_2_O contained in FA and CSA was not included in this ratio. Accordingly, increasing the nominal L/B ratio from 0.35 to 0.45 simultaneously increased the actual W/B ratio from 0.201 to 0.259 and the activator Na_2_O/B ratio from 7.56% to 9.74%. Therefore, the effects associated with increasing L/B reflect the combined influence of water availability and alkaline activator dosage rather than water content alone.

### 2.3. Mixing Procedure

Prior to mixing, the sodium hydroxide solution was prepared by dissolving sodium hydroxide pellets in water to obtain a 12 M solution and allowed to cool to room temperature. The sodium hydroxide and sodium silicate solutions were used at an S/H mass ratio of 1.0 but were added separately during mixing rather than being premixed. Dry materials, including fly ash (FA), calcined seashell ash (CSA), and river sand, were first blended in a pan mixer to ensure a uniform distribution of the binder and aggregate. The sodium hydroxide solution was then gradually added while mixing, followed by the addition of the sodium silicate solution. Mixing continued until a homogeneous mortar was obtained. Immediately after mixing, the fresh mortar was cast into steel molds and externally vibrated for approximately 10 s to ensure proper consolidation and remove entrapped air. The specimens were covered with plastic sheets to minimize moisture loss and stored at room temperature for 24 h. After demolding, the specimens were sealed with plastic film and cured at room temperature until the designated testing ages.

### 2.4. Test Methods

#### 2.4.1. Mini Slump Flow Test

**Figure 1:**
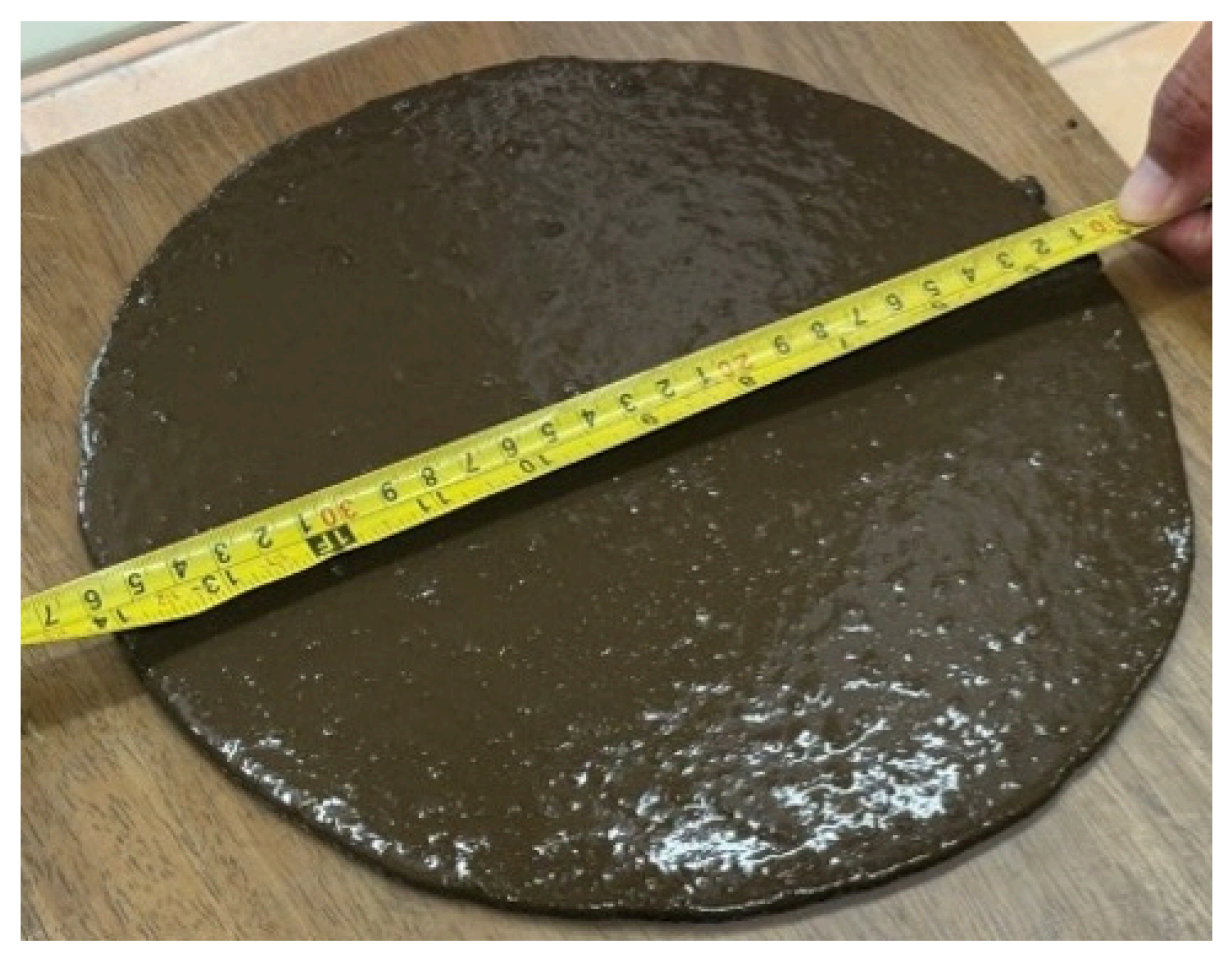
Mini Slump test.

The workability of the fresh alkali-activated mortar was evaluated using a mini-slump flow test. Fresh mortar was placed into a mini-slump cone CONTROLS S.p.A., Liscate, Italy) in two equal layers, with each layer compacted using a tamping rod to minimize entrapped air. After filling, the excess mortar was struck off flush with the top of the cone. The cone was then carefully lifted vertically, allowing the mortar to spread freely under its own weight. The spread diameter was measured in two perpendicular directions, and the average value was reported as the mini-slump flow diameter (mm) (Figure 1). Three specimens were tested for each mixture, and the average value was used for analysis.

#### 2.4.2. Initial Setting Time Test

The initial and final setting times of the alkali-activated binder paste were determined using a Vicat apparatus (Matest, Treviolo (BG), Italy) in accordance with ASTM C191 [19]. The paste mixtures were prepared using the same binder composition, alkaline activator concentration, and liquid-to-binder ratio as the corresponding mortar mixtures, but without the addition of river sand.

Immediately after mixing, the fresh paste was placed into the Vicat mold and the surface was leveled. The penetration of a 1 mm diameter Vicat needle was measured at regular time intervals in accordance with ASTM C191 [19]. The initial setting times were determined based on the penetration criteria specified in the standard. Three specimens were tested for each mixture, and the average values were reported.

#### 2.4.3. Compressive Strength

The compressive strength of the alkali-activated mortars was determined in accordance with ASTM C109/C109M [20] using 50 mm × 50 mm × 50 mm cube specimens. After casting, the specimens were sealed and cured at room temperature until the designated testing ages of 28 days. Prior to testing, the specimen surfaces were cleaned to remove any loose particles. The specimens were then tested using a Universal Testing Machine (UTM) (NL Scientifics Instrument Sdn. Bhd., Selangor, Malaysia) under a constant loading rate in accordance with ASTM C109/C109M [20] until failure. Three specimens were tested for each mixture and curing age, and the average compressive strength was reported.

#### 2.4.4. Flexural Strength

The flexural strength of the alkali-activated mortars was determined in accordance with ASTM C348 [21] using prism specimens measuring 40 mm × 40 mm × 160 mm. After casting, the specimens were sealed and cured at room temperature until the designated testing ages of 28 days. Flexural testing was conducted using a Universal Testing Machine (UTM) (NL Scientifics Instrument Sdn. Bhd., Selangor, Malaysia) equipped with a three-point bending fixture. The load was applied continuously at the loading rate specified in ASTM C348 [21] until specimen failure. Three specimens were tested for each mixture and curing age, and the average flexural strength was reported.

#### 2.4.5. Accelerated Carbonation Exposure

**Figure 2:**
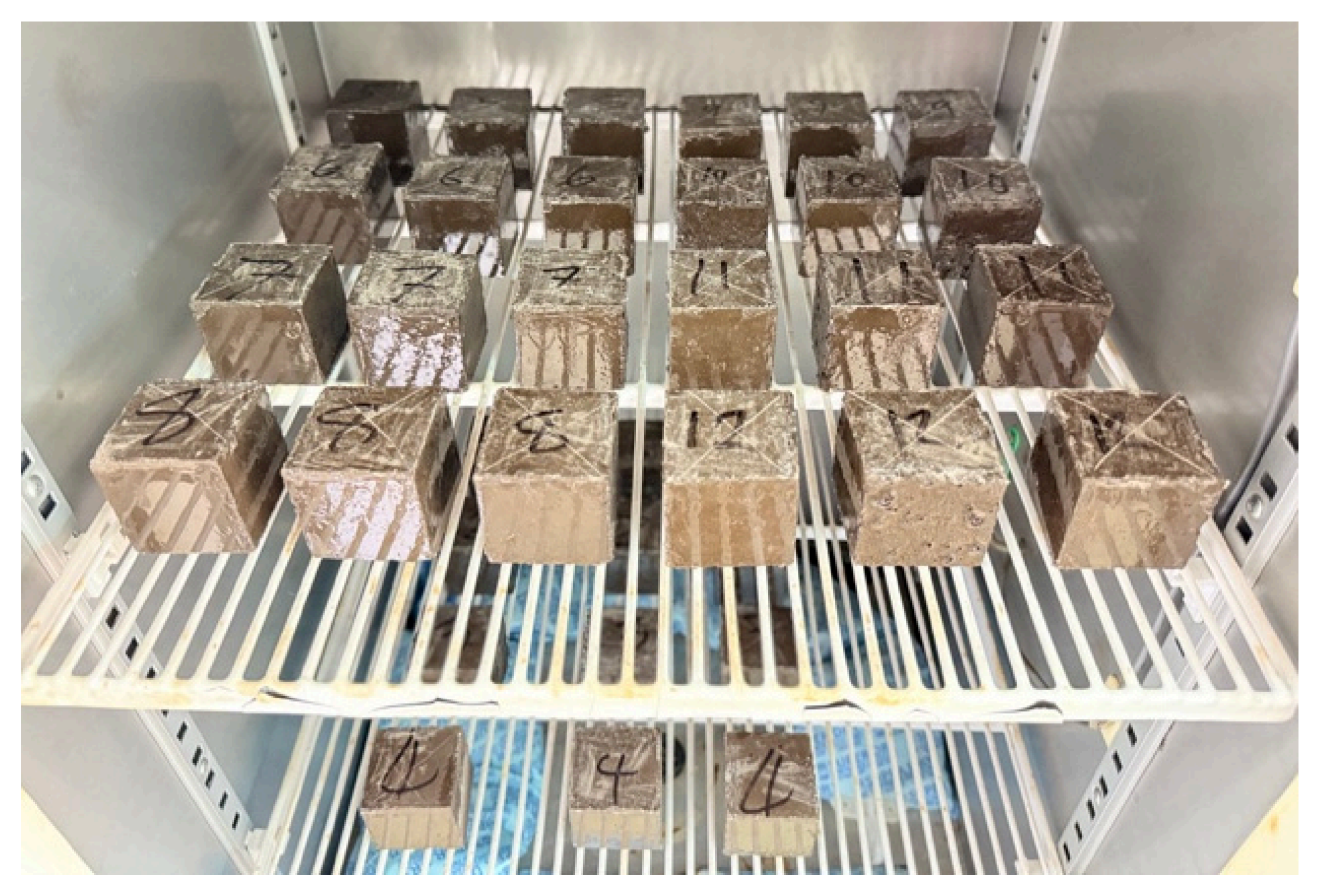
Carbonation test.

After curing at room temperature for 28 days, 50 mm × 50 mm × 50 mm cubic specimens were subjected to accelerated carbonation. Prior to carbonation exposure, five surfaces of each specimen were sealed with epoxy resin, leaving only one vertical surface exposed to CO_2_ (Figure 2). This configuration was adopted to restrict CO_2_ ingress predominantly to one direction and provide a well-defined carbonation front for subsequent carbonation-depth measurement.

The prepared specimens were placed in an accelerated carbonation chamber (C.B.N. Materialtest Co., Ltd., Bangkok, Thailand) maintained at a CO_2_ concentration of 5%, a temperature of 20 ± 2 °C, and a relative humidity of 65 ± 5%. The CO_2_ concentration of 5% was selected to accelerate carbonation within the experimental exposure period and is consistent with conditions previously employed for accelerated carbonation testing of alkali-activated materials [22,23]. However, because the elevated CO_2_ concentration differs substantially from atmospheric exposure and may influence carbonation kinetics and reaction processes, the results obtained under these conditions should be interpreted as accelerated carbonation responses rather than as direct representations of natural carbonation behavior.

The specimens were exposed under these conditions for 28 days. In parallel, the corresponding non-carbonated control specimens were wrapped with plastic film and stored under controlled temperature and humidity conditions for the same 28-day period. Consequently, both the carbonated and non-carbonated specimens were 56 days old at the time of subsequent characterization.

#### 2.4.6. Carbonation Depth

For the carbonation investigation, G0 and G20 were selected to represent the two endpoints of CSA incorporation, corresponding to 0% and 20% CSA replacement, respectively. This selection provided the greatest contrast in CSA content for evaluating its influence on carbonation behavior. Accordingly, the carbonation results represent a direct comparison between G0 and G20 and should not be interpreted as establishing a continuous response with increasing CSA replacement level.

Following 28 days of accelerated carbonation exposure, each cubic specimen was split perpendicular to the exposed surface to produce a fractured cross-section extending from the exposed surface toward the interior of the specimen. Loose particles and dust were removed from the fractured surface without the use of water.

A phenolphthalein indicator solution was then sprayed uniformly onto the freshly exposed surface. Areas retaining sufficient alkalinity developed a pink-to-purple coloration, whereas carbonated or substantially neutralized regions remained colorless. The carbonation front was identified as the boundary between the colored and colorless regions.

The carbonation depth was measured from the exposed surface toward the interior of the specimen at several evenly distributed locations along the fractured cross-section, avoiding corners and visibly damaged areas. Measurements were taken using a digital caliper or image-analysis software with an appropriate dimensional scale. The average carbonation depth was calculated from the individual measurements obtained along the exposed surface.

For each mixture, the reported carbonation depth represents the mean value obtained from three replicate specimens. The variability of the measurements was expressed using the standard deviation. Because alkali-activated materials may exhibit partial alkalinity reduction without a sharply defined phenolphthalein boundary, the indicator results were interpreted as the depth of alkalinity loss rather than as a direct measurement of the total amount of carbon dioxide chemically bound by the mortar.

#### 2.4.7. X-Ray Diffraction Analysis (XRD)

X-ray diffraction (XRD) was performed to investigate the mineralogical composition of the alkali-activated mortars before and after accelerated carbonation. Following carbonation exposure, the specimens were wrapped in plastic film until sample preparation, and no additional drying treatment was applied. For the carbonated specimens, each specimen was split into two halves. One half was sprayed with phenolphthalein solution to determine the carbonation depth. Based on the measured carbonation depth, material corresponding to the carbonated zone was collected from the other half, which had not been exposed to phenolphthalein. Thus, the sampling depth corresponded to the measured carbonation depth of each specimen rather than to a fixed depth. The material from the identified carbonated zone was ground into fine powder, from which the sample for XRD analysis was collected without preferential selection and stored in a sealed plastic bag until the date of testing. XRD patterns were recorded over a 2θ range of 20–70°. Crystalline phase identification and diffraction-pattern fitting were performed using GSAS-II software version 5.6.0 (Argonne National Laboratory, Lemont, IL, USA) with crystal structure models obtained from the American Mineralogist Crystal Structure Database (AMCSD), including calcite (AMCSD 0000098), quartz (AMCSD 0006212), anorthite (AMCSD 0020641), and Mullite (AMCSD 0006888). The resulting diffraction patterns were used to identify the crystalline phases present and to qualitatively assess changes in carbonate-bearing phases associated with accelerated carbonation.

#### 2.4.8. Thermogravimetric Analysis (TGA)

Thermogravimetric analysis (TGA) was conducted to evaluate the temperature-dependent mass-loss behavior of the alkali-activated mortars and to identify changes associated with carbonation. Approximately 20 mg of each sample was placed in a crucible and heated from 30 to 900 °C at a constant heating rate of 10 °C/min under a nitrogen (N_2_) atmosphere with a gas flow rate of 20 mL/min. The sample mass was continuously recorded as a function of temperature, and the corresponding derivative thermogravimetric (DTG) curves were obtained to facilitate identification of the principal mass-loss regions. Mass losses were evaluated over selected temperature intervals based on changes in the TG curves and the corresponding DTG features. Particular attention was given to the carbonate-associated decomposition region, and the mass-loss behavior before and after accelerated carbonation was compared to assess changes associated with mineral carbonation.

## 3. Results and Discussion

### 3.1. Spread Flow

**Figure 3:**
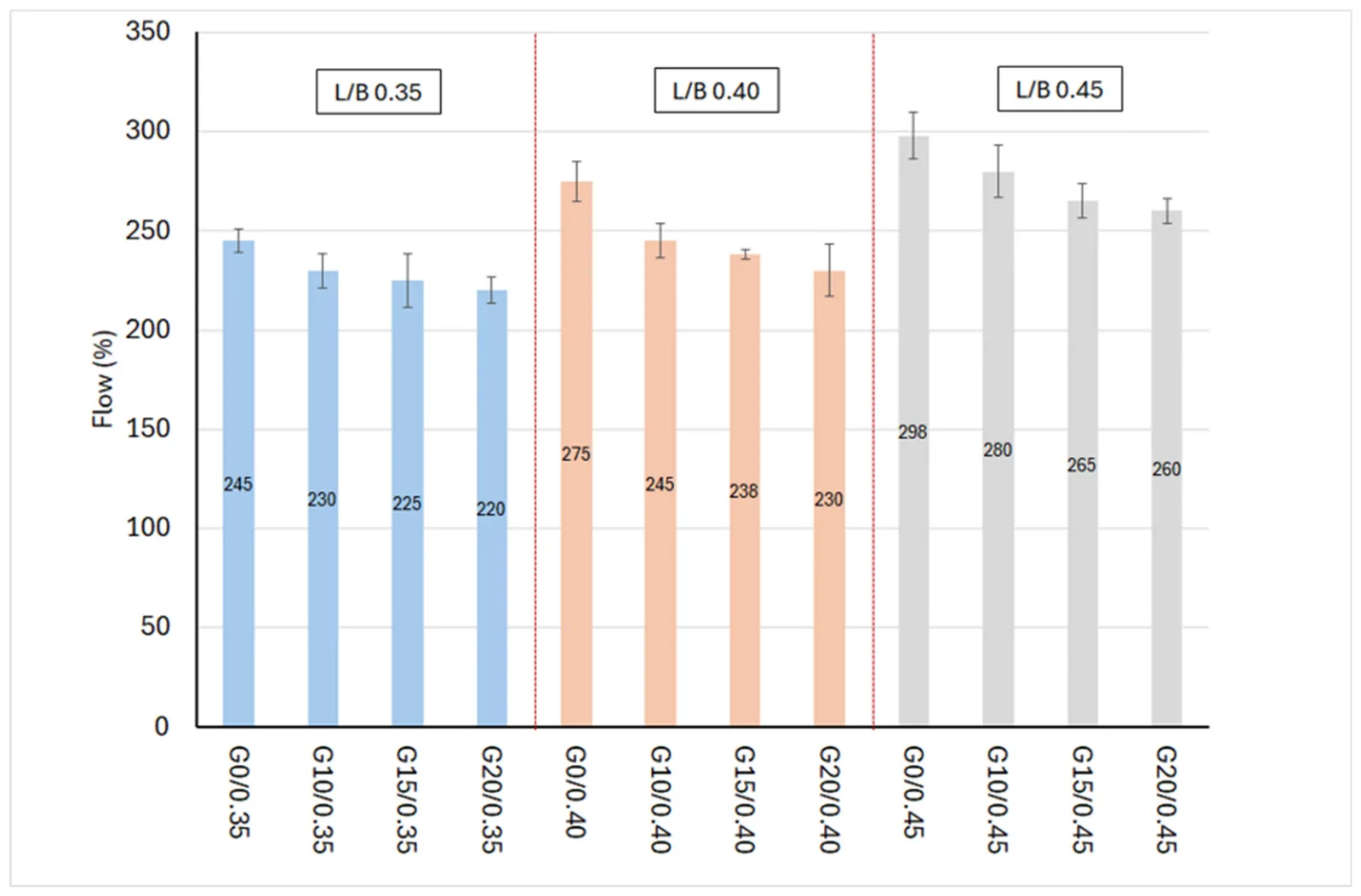
Spread flow of alkali-activated material.

Figure 3 presents the spread flow of the alkali-activated mortars as a function of liquid-to-binder (L/B) ratio and CSA replacement level. The results show two clear overall trends: increasing the L/B ratio increased the spread flow, whereas increasing the CSA content generally reduced it. Across all mixtures, the spread flow ranged from 220 to 298 mm, indicating that all mixtures maintained adequate workability for casting.

For the G0 mixtures, increasing the L/B ratio from 0.35 to 0.45 increased the spread flow from 245 to 298 mm, corresponding to an increase of approximately 21.6%. Similar increases with L/B ratio were observed for the CSA-containing mixtures. Because the L/B ratio was varied by proportionally increasing both the NaOH and sodium silicate solutions, the higher L/B ratios provided a greater amount of alkaline activator solution relative to the solid binder while maintaining the same activator composition. The increased solution volume improves particle lubrication, reduces interparticle friction, and facilitates movement of the solid particles during flow. Therefore, the observed increase in spread flow reflects the increased alkaline activator dosage relative to the binder rather than an increase in liquid content alone.

In contrast, CSA incorporation generally reduced the spread flow at each L/B ratio. At L/B = 0.35, the spread flow decreased from 245 mm for G0 to 220 mm for G20, corresponding to a reduction of approximately 10.2%. The reduction was more pronounced at L/B = 0.40, where the spread flow decreased from 275 to 230 mm, or approximately 16.4%. At L/B = 0.45, the corresponding decrease was from 298 to 260 mm, representing a reduction of approximately 12.8%. Although some differences between adjacent CSA replacement levels were relatively small compared with the experimental variability indicated by the error bars, the overall trend demonstrates that higher CSA incorporation reduced the flowability of the mortar.

The reduction in spread flow may be associated with both physical and chemical effects of CSA incorporation. The CSA increased the liquid demand of the binder system, thereby reducing the amount of free liquid available for particle lubrication. In addition, the high calcium content of CSA and the presence of potentially reactive Ca-bearing phases may have promoted more rapid early reactions and structural build-up within the fresh alkali-activated binder. Calcium-rich constituents have previously been reported to accelerate early reaction and setting in alkali-activated systems [24,25]. The progressive reduction in spread flow with CSA incorporation is therefore consistent with the increased availability of reactive calcium and the initial-setting behavior discussed in Section 3.2.

### 3.2. Setting Time

**Figure 4:**
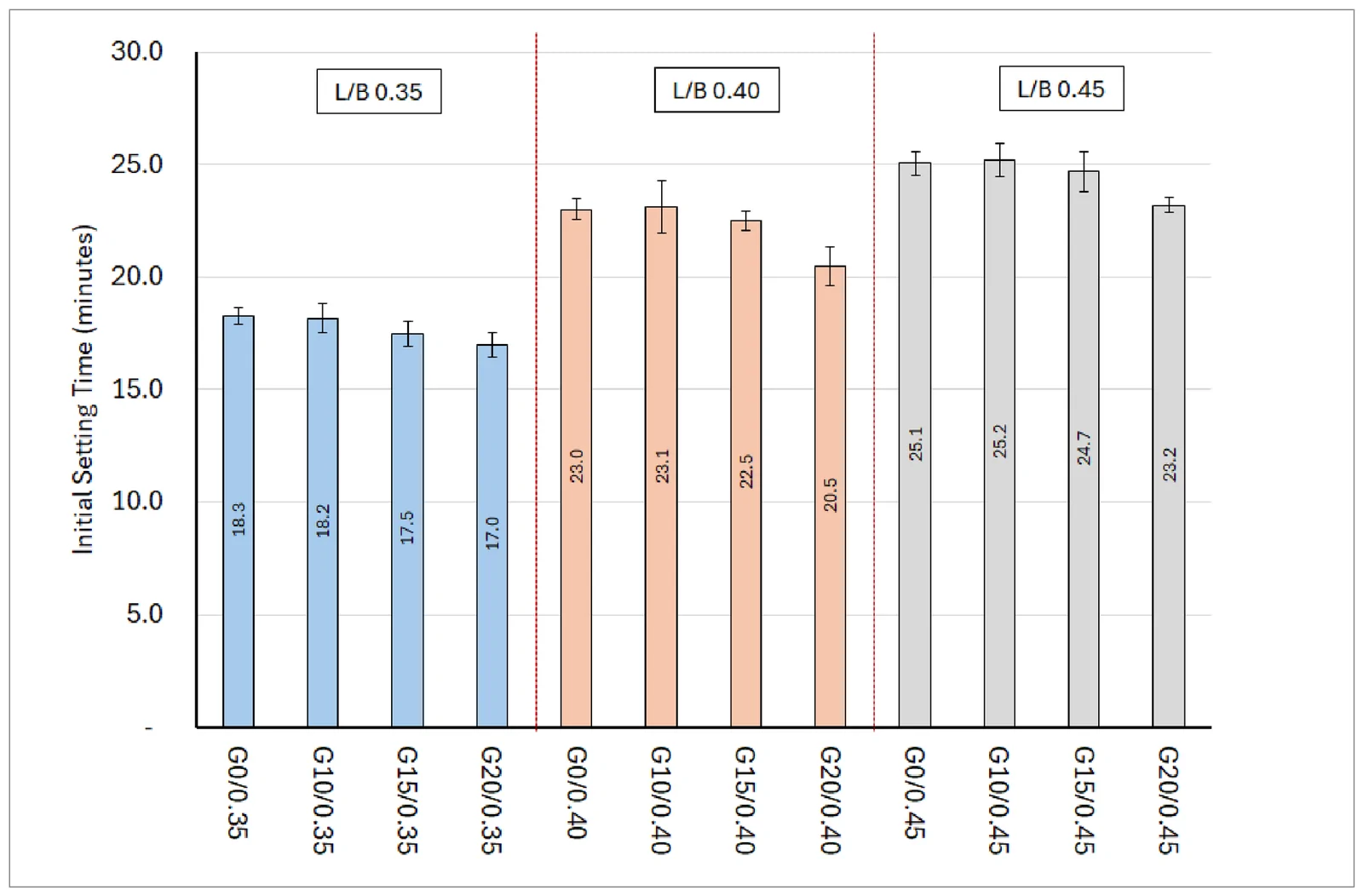
Initial setting time of alkali-activated material.

Figure 4 presents the initial setting times of the alkali-activated pastes as functions of the liquid-to-binder (L/B) ratio and CSA replacement level. Overall, increasing the L/B ratio prolonged the initial setting time. The G0 mixture exhibited an initial setting time of approximately 18 min at L/B = 0.35, increasing to approximately 23 min at L/B = 0.40 and 25 min at L/B = 0.45. This trend demonstrates that the L/B ratio strongly influenced the early setting behavior of the alkali-activated binder.

The longer initial setting time at higher L/B ratios can be attributed to the greater amount of alkaline activator solution relative to the binder. Because the L/B ratio was increased by proportionally increasing both the NaOH and sodium silicate solutions, the higher L/B ratios increased both the solution volume and the alkaline activator dosage relative to the binder. The greater solution volume increases the separation between solid particles and provides a larger volume of free liquid within the paste, thereby delaying the development of sufficient rigidity to reach the initial setting condition. At the same time, the increased activator dosage may influence precursor dissolution and early reaction-product formation. Conversely, at the lower L/B ratio, the reduced solution volume promotes closer particle interaction and more rapid structural build-up, resulting in shorter initial setting times.

The effect of CSA was dependent on the L/B ratio. At L/B = 0.35, increasing the CSA content produced only a relatively small reduction in initial setting time. This limited effect may be attributed to the already rapid setting of the low-L/B paste, leaving less opportunity for additional CSA to further accelerate the initial structural development. At L/B = 0.40, the accelerating effect became more apparent, particularly at the higher CSA replacement level, with G20 showing the shortest initial setting time within this group. A similar but clearer response was observed at L/B = 0.45. Although G10 produced little change relative to G0, further increasing the CSA content progressively shortened the initial setting time, with G20 exhibiting the lowest value.

The reduction in initial setting time at higher CSA contents can be associated with both physical and chemical effects. The incorporation of CSA increased the demand for liquid, consistent with the reduction in spread flow observed in Section 3.1, thereby reducing the amount of free liquid available for particle movement. More importantly, the high calcium content of CSA indicates the presence of additional Ca-bearing constituents that may participate in the alkali-activation process and promote early precipitation of Ca-containing reaction products alongside the aluminosilicate reaction products of the fly ash-based system [25]. These processes can accelerate early structural build-up and consequently reduce the time required to reach initial set.

Overall, the results indicate an interaction between L/B ratio and CSA content in controlling initial setting. At low L/B ratio, rapid setting associated with the low liquid content appears to dominate the response, and the additional accelerating effect of CSA is comparatively small. At higher L/B ratios, where the greater liquid content delays initial structural development, the accelerating influence of CSA becomes more evident, particularly at the higher replacement levels. This behavior suggests that the effect of CSA on setting is governed by the balance between liquid availability and the enhanced early reaction associated with the additional Ca-rich component.

### 3.3. Mechanical Properties

#### 3.3.1. Compressive Strength

**Figure 5:**
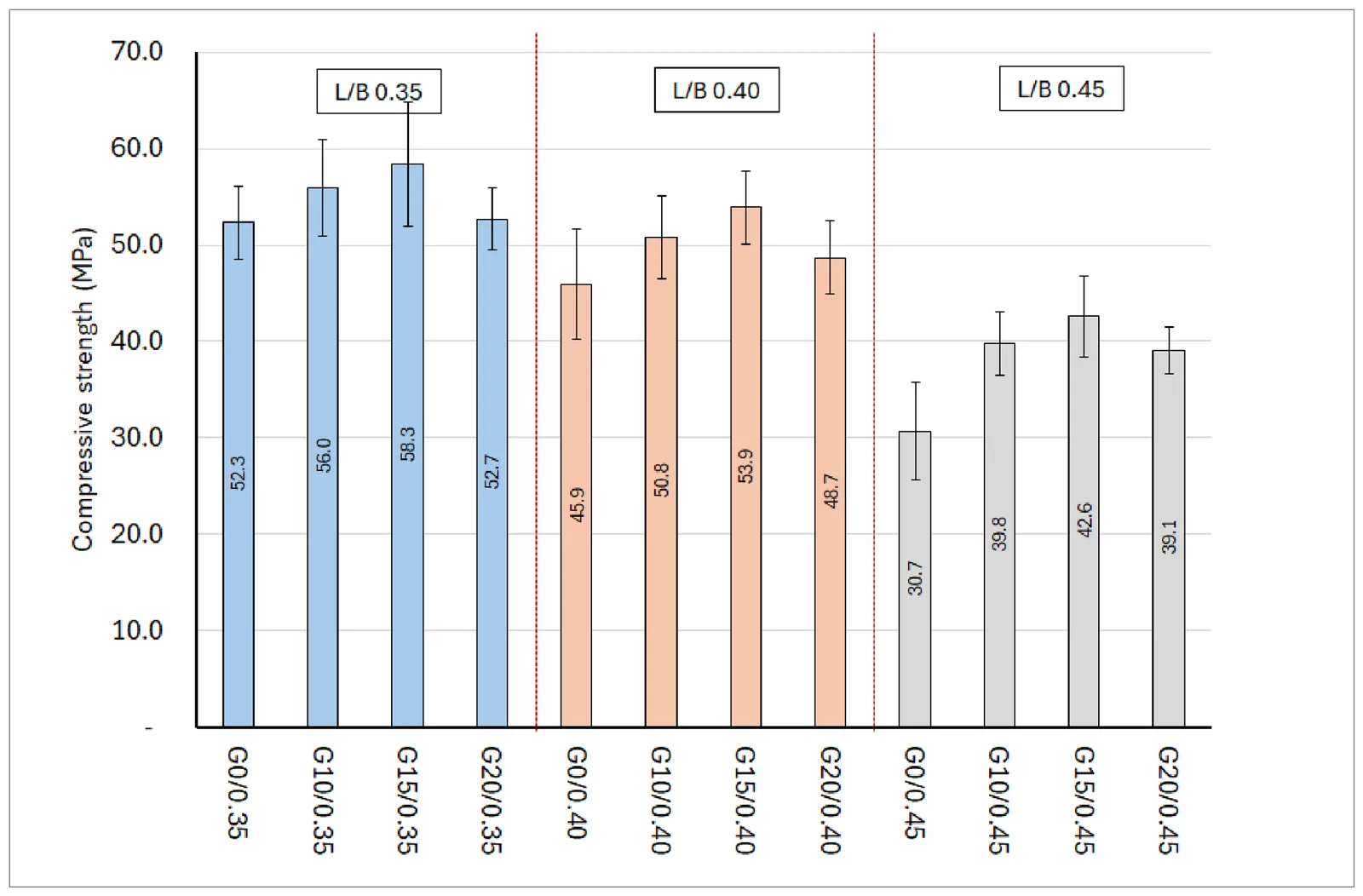
Compressive strength of alkali-activated material.

Figure 5 presents the 28-day compressive strength of the alkali-activated mortars as a function of liquid-to-binder (L/B) ratio and CSA replacement level. Increasing the L/B ratio consistently reduced the compressive strength of all mixtures. For the control mixture (G0), the compressive strength decreased from 52.3 MPa at L/B = 0.35 to 45.9 MPa at L/B = 0.40 and 30.7 MPa at L/B = 0.45, corresponding to an overall reduction of approximately 41.3%. Similar reductions were observed for the G10, G15, and G20 mixtures, confirming the strong influence of L/B ratio on the mechanical performance of the alkali-activated mortar.

The reduction in compressive strength with increasing L/B ratio can be attributed to the greater amount of alkaline activator solution relative to the binder. Because both the NaOH and sodium silicate solution contents increased with L/B ratio, the higher L/B ratios increased not only the solution volume but also the alkaline activator dosage relative to the binder. Although the greater solution volume improved the workability of the fresh mortar, it may result in a greater volume of residual pores after hardening and a less compact reaction-product network. In addition, the increased activator dosage may alter precursor dissolution and reaction-product formation. These combined effects can contribute to the reduction in load-bearing capacity at higher L/B ratios. Similar reductions in compressive strength with increasing liquid-to-binder ratio have been reported for high-calcium fly ash-based geopolymer systems [26].

CSA incorporation generally improved the compressive strength up to a replacement level of 15%. At L/B = 0.35, the strength increased from 52.3 MPa for G0 to 56.0 and 58.3 MPa for G10 and G15, respectively. Similar trends were observed at L/B = 0.40, where the strength increased from 45.9 to 50.8 and 53.9 MPa. The improvement was particularly pronounced at L/B = 0.45, with strength increasing from 30.7 MPa for G0 to 39.8 MPa for G10 and 42.6 MPa for G15. Thus, G15 exhibited the highest mean compressive strength within each L/B group. However, the experimental variability indicated by the error bars should be considered when comparing mixtures with relatively small differences in mean strength.

The strength enhancement associated with CSA incorporation may be related to its high calcium content and the presence of Ca-bearing constituents capable of participating in the alkali-activation process. These Ca-bearing species may promote the formation of calcium-containing reaction products alongside the aluminosilicate gel network produced through fly ash activation. Previous studies have shown that calcium-bearing constituents can modify reaction-product formation in alkali-activated fly ash systems and promote the coexistence of calcium-containing and aluminosilicate-type gels, which can contribute to mechanical strength development [27,28]. This interpretation is also consistent with the reduction in spread flow and shorter initial setting time observed following CSA incorporation, indicating more rapid early structural development of the binder [20].

Increasing the CSA replacement from 15% to 20% reduced the mean compressive strength at all three L/B ratios. The strengths decreased from 58.3 to 52.7 MPa at L/B = 0.35, from 53.9 to 48.7 MPa at L/B = 0.40, and from 42.6 to 39.1 MPa at L/B = 0.45. At higher replacement levels, the additional calcium supplied by CSA is accompanied by a reduction in the amount of fly ash available for alkali activation. Consequently, the beneficial effect of additional calcium may eventually be offset by the reduced availability of aluminosilicate precursors and changes in the balance of Ca-, Si-, and Al-bearing species involved in reaction-product formation. Similar optimum ranges have been reported for Ca-rich additions to fly ash-based alkali-activated systems [29].

Notably, the relative strength benefit associated with CSA became greater as the L/B ratio increased. The G15 mixture exhibited mean compressive-strength increases of approximately 11.5%, 17.4%, and 38.8% relative to G0 at L/B ratios of 0.35, 0.40, and 0.45, respectively. The larger relative improvement at L/B = 0.45 suggests that the contribution of CSA became increasingly important as the baseline strength of the fly ash-based matrix decreased. Therefore, the mechanical response appears to be governed by an interaction between liquid content and CSA incorporation rather than by either parameter independently.

#### 3.3.2. Flexural Strength

**Figure 6:**
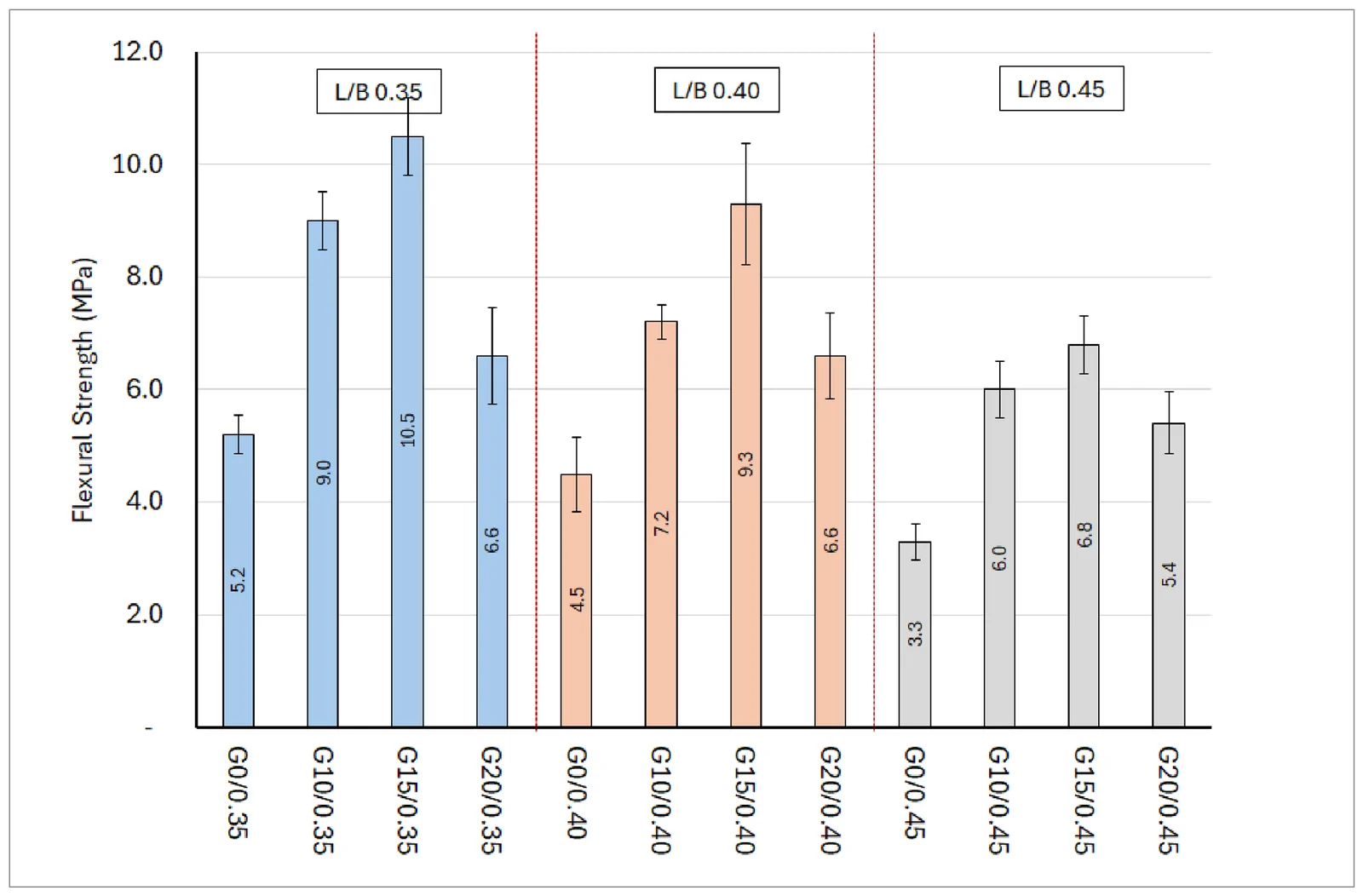
Flexural strength of alkali-activated material.

Figure 6 presents the 28-day flexural strength of the fly ash-based alkali-activated mortars as a function of liquid-to-binder (L/B) ratio and CSA replacement level. The flexural-strength response was influenced by both parameters. In general, increasing the L/B ratio reduced the flexural strength, whereas CSA incorporation substantially improved the strength up to a replacement level of 15%. For the control mixture, the flexural strength decreased from 5.2 MPa at L/B = 0.35 to 4.5 MPa at L/B = 0.40 and 3.3 MPa at L/B = 0.45, corresponding to an overall reduction of approximately 36.5%. Similar reductions with increasing L/B ratio were generally observed for the CSA-containing mixtures.

The reduction in flexural strength with increasing L/B ratio may be associated with changes in the hardened matrix resulting from the greater amount of alkaline activator solution relative to the binder. Because both the NaOH and sodium silicate solution contents increased with L/B ratio, the higher L/B ratios increased not only the solution volume but also the alkaline activator dosage relative to the binder. Although the greater solution volume improved the workability of the fresh mortar, it may result in greater residual pore volume after hardening and a less compact reaction-product network. In addition, the increased activator dosage may alter precursor dissolution and reaction-product formation. These combined effects can reduce the ability of the matrix to resist tensile stresses generated during bending. Previous studies have similarly reported that liquid content and the resulting pore characteristics can strongly influence the mechanical performance of alkali-activated materials [30,31].

CSA incorporation produced a pronounced improvement in flexural strength. At L/B = 0.35, the strength increased from 5.2 MPa for G0 to 9.0 MPa for G10 and reached 10.5 MPa for G15. Similar trends were observed at L/B = 0.40, where the strength increased from 4.5 to 7.2 and 9.3 MPa, and at L/B = 0.45, where it increased from 3.3 to 6.0 and 6.8 MPa. Relative to G0, G15 increased the mean flexural strength by approximately 102%, 107%, and 106% at L/B ratios of 0.35, 0.40, and 0.45, respectively. G15 therefore exhibited the highest mean flexural strength within each L/B group. The magnitude and consistency of these increases, together with the experimental variability shown by the error bars, demonstrate a clear beneficial effect of CSA incorporation within the investigated replacement range.

The improvement in flexural strength may be associated with the Ca-rich constituents introduced through CSA incorporation. Previous studies have shown that seashell-derived and other Ca-rich constituents can modify reaction-product formation in alkali-activated systems and contribute to improved mechanical performance [32,33,34]. In the present system, Ca-bearing species derived from CSA may have participated in the formation of calcium-containing reaction products alongside the aluminosilicate gel network, thereby improving the integrity and bonding of the hardened matrix. This interpretation is consistent with the increased compressive strength and the shorter initial setting time observed following CSA incorporation, although direct characterization of the gel structure would be required to confirm the specific mechanism.

Increasing the CSA replacement level from 15% to 20% resulted in a clear reduction in flexural strength at all L/B ratios. The strength decreased from 10.5 to 6.6 MPa at L/B = 0.35, from 9.3 to 6.6 MPa at L/B = 0.40, and from 6.8 to 5.4 MPa at L/B = 0.45, corresponding to reductions of approximately 37.1%, 29.0%, and 20.6%, respectively. At the higher replacement level, the increased proportion of the Ca-rich CSA component was accompanied by a greater reduction in the amount of fly ash available for alkali activation. The balance between the Ca-rich component and aluminosilicate precursor therefore appears important for flexural performance, with 15% CSA providing the highest mean strength within the investigated range.

Notably, the relative improvement in flexural strength associated with G15 was considerably greater than the corresponding improvement in compressive strength. G15 increased the mean compressive strength by approximately 11.5–38.8%, whereas the flexural strength increased by approximately 102–107%. This significant improvement in flexural strength may be associated with microstructural refinement and enhanced interfacial bonding resulting from the presence of Ca-rich reaction products. Previous studies have indicated that seashell-derived and Ca-rich constituents can promote matrix densification and improve interfacial characteristics in alkali-activated systems [27,28], which may contribute to greater resistance to crack initiation and propagation under flexural loading. The different magnitude of improvement should also be considered in relation to the respective testing modes. Under flexural loading, failure is particularly sensitive to tensile crack initiation, local defects, and interfacial characteristics, whereas compressive strength reflects the overall load-bearing response of the matrix [35,36]. In the present results, the substantially greater relative improvement in flexural strength was consistently observed at all three L/B ratios, suggesting that the beneficial effect associated with G15 was particularly pronounced under the crack-sensitive bending condition. This difference indicates that the influence of CSA was particularly pronounced under bending rather than representing a proportional increase in all mechanical properties.

Although the present results do not directly quantify fracture toughness or crack-growth resistance, the substantial improvement in flexural strength demonstrates that an appropriate CSA content can significantly enhance the bending performance of the fly ash-based alkali-activated mortar.

### 3.4. Carbonation Depth

**Figure 7:**
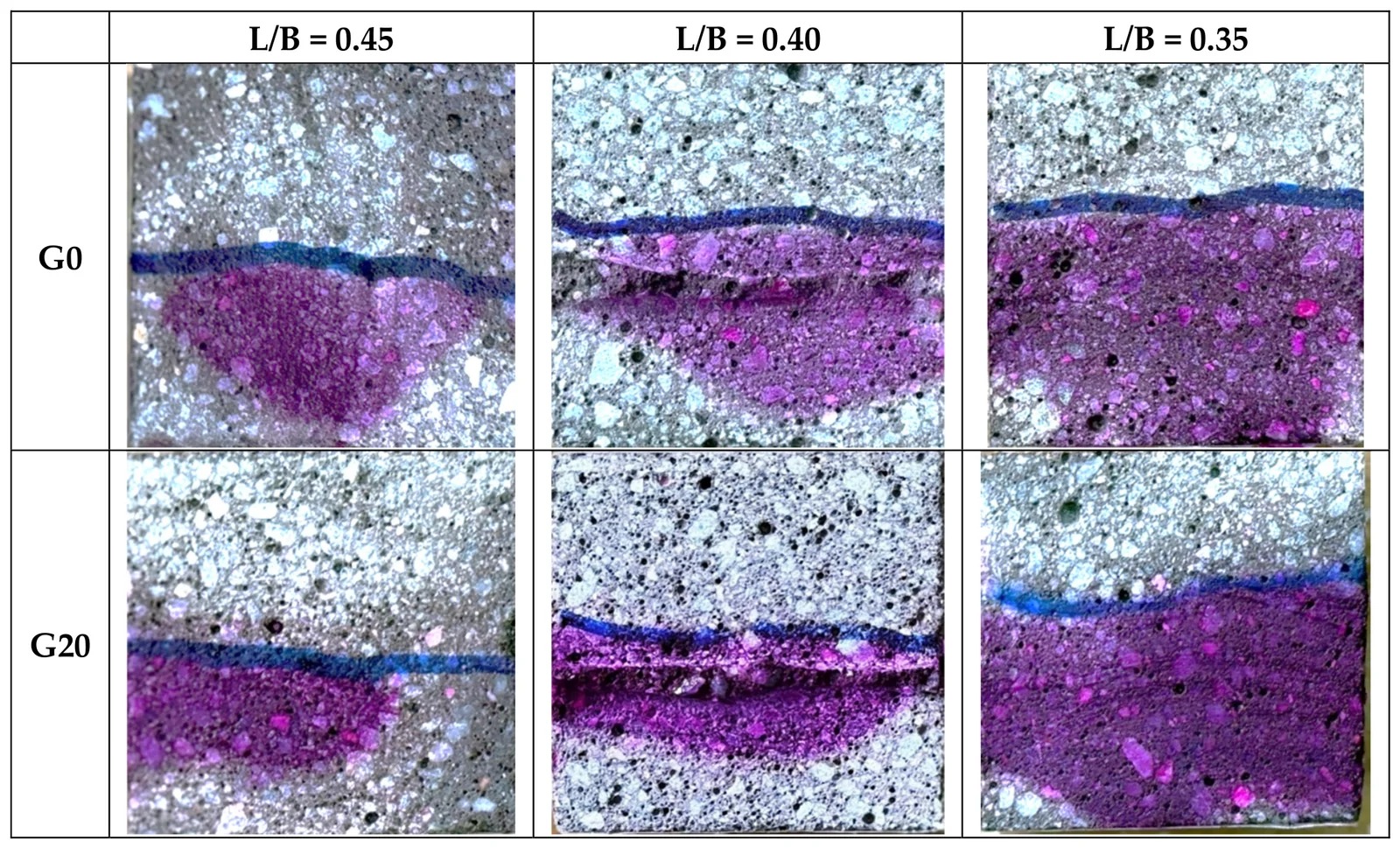
Representative phenolphthalein-stained cross-sections of G0 and G20 alkali-activated mortars after 28 days of accelerated carbonation at L/B ratios of 0.35, 0.40, and 0.45. The blue lines indicate the identified carbonation fronts between the colorless carbonated/alkalinity-reduced region and the purple alkaline region.

**Figure 8:**
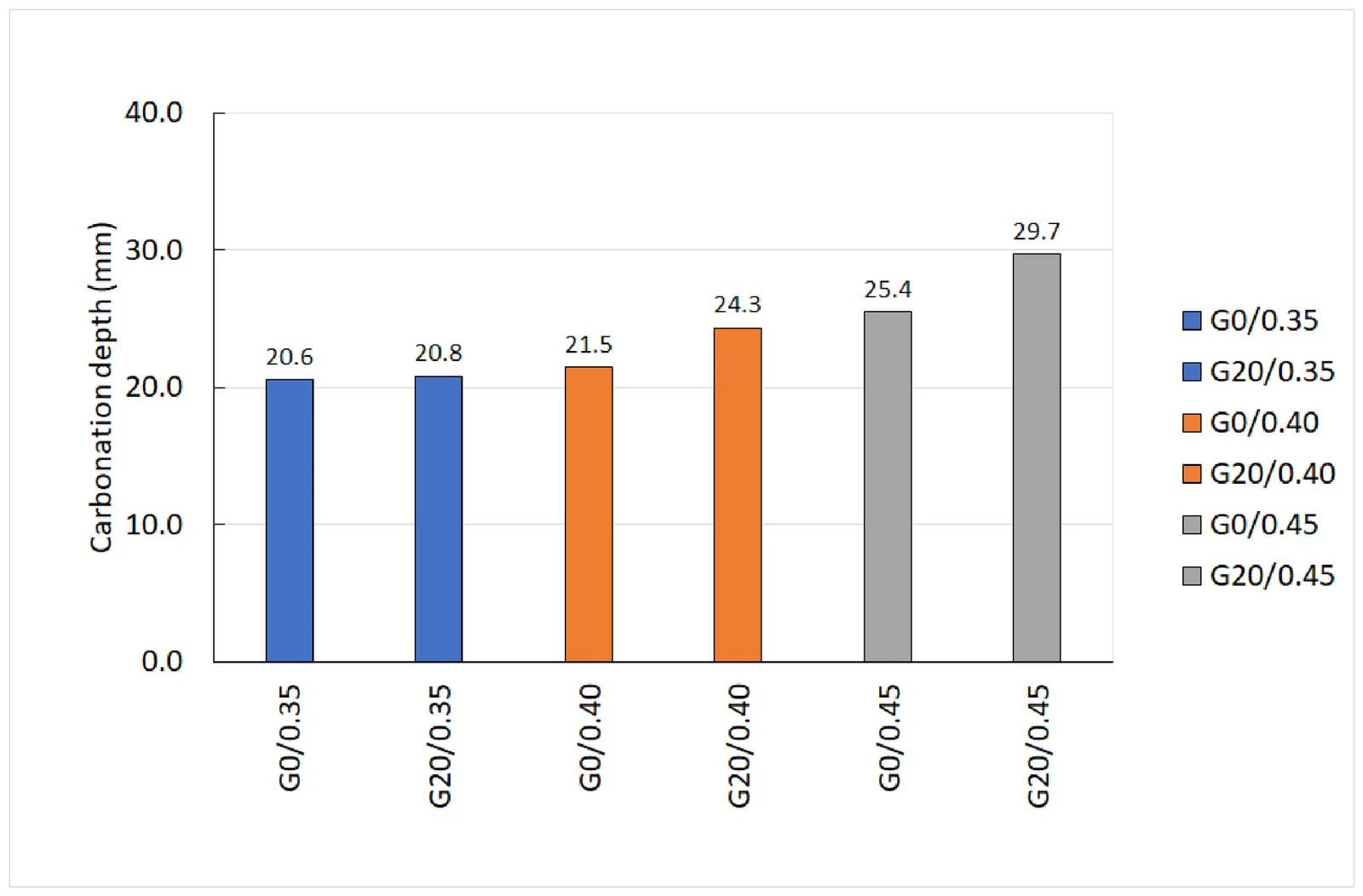
Carbonation depths of G0 and G20 alkali-activated mortars after 28 days of accelerated carbonation at different L/B ratios. Values represent the mean ± standard deviation of three replicate specimens.

Figure 7 presents representative phenolphthalein-stained cross-sections of the G0 and G20 mortars following 28 days of accelerated carbonation, while Figure 8 summarizes the corresponding carbonation depths. The colorless region represents the carbonated zone, whereas the purple region indicates the portion of the matrix retaining sufficient alkalinity to produce the phenolphthalein response. The photographs show a progressive increase in the depth of the colorless region with increasing liquid-to-binder (L/B) ratio, particularly for the G20 mixtures.

For the G0 mixtures, the carbonation depth increased from 20.56 mm at L/B = 0.35 to 21.47 mm at L/B = 0.40 and 25.44 mm at L/B = 0.45. Thus, increasing the L/B ratio from 0.35 to 0.45 resulted in an overall increase of 4.88 mm, or approximately 23.7%, in carbonation depth. A stronger dependence on L/B ratio was observed for the G20 mixtures, for which the carbonation depth increased from 20.80 mm at L/B = 0.35 to 24.31 mm at L/B = 0.40 and 29.67 mm at L/B = 0.45. The corresponding increase from L/B = 0.35 to 0.45 was 8.87 mm, equivalent to approximately 42.6%. These results demonstrate that increasing L/B ratio promoted deeper progression of the carbonation front in both binder systems.

The influence of CSA was strongly dependent on the L/B ratio. At L/B = 0.35, the carbonation depths of G0 and G20 were nearly identical at 20.56 and 20.80 mm, respectively, representing a difference of only 0.24 mm or approximately 1.2%. This small difference is within the experimental variability and therefore does not indicate an appreciable effect of CSA on carbonation depth at the lowest L/B ratio. At L/B = 0.40, however, incorporation of 20% CSA increased the carbonation depth from 21.47 to 24.31 mm, corresponding to an increase of 2.84 mm or approximately 13.2%. At L/B = 0.45, the effect became still more pronounced, with carbonation depth increasing from 25.44 mm for G0 to 29.67 mm for G20, an increase of 4.23 mm or approximately 16.6%. The gradually greater difference between G0 and G20 with increasing L/B ratio is also evident from the phenolphthalein-stained cross-sections in Figure 7.

This interaction between L/B ratio and CSA incorporation may be associated with the combined influence of CO_2_ accessibility and the availability of reactive Ca-bearing species. At the lower L/B ratio, the lower liquid content and substantially higher compressive strength are consistent with matrix characteristics that may have provided greater resistance to CO_2_ ingress. Under these conditions, restricted CO_2_ accessibility could have limited interaction with the additional Ca-bearing phases supplied by CSA. This interpretation is consistent with the nearly identical carbonation depths measured for G0/0.35 and G20/0.35, although pore structure and CO_2_ transport were not directly characterized in the present study.

As the L/B ratio increased, the reduction in mechanical strength may indicate matrix characteristics more favorable to CO_2_ penetration. Greater CO_2_ accessibility could allow the carbonation front to progress further into the mortar and increase the opportunity for dissolved CO_2_ species to interact with reactive Ca-bearing phases. Under these conditions, the additional Ca supplied by CSA could participate more effectively in carbonation reactions, providing a plausible explanation for why the difference between G0 and G20 increased from approximately 1.2% at L/B = 0.35 to 13.2% at L/B = 0.40 and 16.6% at L/B = 0.45. These proposed transport-related mechanisms remain hypotheses because porosity, permeability, and CO_2_ diffusivity were not directly measured.

This finding is important in the context of engineered CO_2_ mineralization. Increasing the availability of Ca-bearing phases alone may not necessarily enhance mineral carbonation when CO_2_ transport through the matrix is strongly restricted. Rather, effective mineralization requires both a source of reactive calcium and sufficient accessibility of CO_2_ within the hardened matrix. The carbonation-depth results therefore provide macroscopic evidence of the interaction between these two factors, while the XRD and TG/DTG analyses discussed subsequently provide complementary evidence regarding the formation of carbonate-bearing phases.

It should be emphasized, however, that the phenolphthalein method identifies the progression of alkalinity loss rather than directly measuring the amount of CO_2_ chemically bound within the mortar. Consequently, a greater carbonation depth should not by itself be interpreted as proportionally greater CO_2_ uptake. The carbonation-depth results are therefore considered together with the mineralogical and thermal analyses to evaluate the enhanced mineral carbonation associated with CSA incorporation.

To further quantify the influence of CSA on carbonation progression, a carbonation-depth enhancement factor (*CEF_x_*) was defined for comparison by normalizing the increase in carbonation depth of the CSA-containing mixture relative to the corresponding control mixture after 28 days of accelerated carbonation:

(1) $$\mathrm{CE}F_{x}=\frac{x_{\mathrm{CSA}}-x_{G0}}{x_{G0}}\times 100$$

where *x*_CSA_ is the measured carbonation depth of the CSA-containing mixture and *x*_G0_ is the measured carbonation depth of the corresponding control mixture without CSA, both determined at the same L/B ratio and under the same carbonation exposure conditions. A positive *CEF_x_* therefore represents an increase in carbonation depth associated with CSA incorporation relative to the corresponding G0 mixture. The calculated *CEF_x_* values are specific to the 28-day accelerated carbonation condition employed in this study.

As summarized in Table 3, *CEF_x_* was only 1.2% at L/B = 0.35, indicating that CSA incorporation produced little change in carbonation depth under the low-L/B condition. However, *CEF_x_* increased markedly to 13.2% at L/B = 0.40 and further to 16.6% at L/B = 0.45. Because *CEF_x_* is normalized against the corresponding G0 mixture at each L/B ratio, this trend indicates that the relative contribution of CSA to carbonation progression became increasingly pronounced as the L/B ratio increased.

This behavior suggests an interaction between CSA-derived Ca availability and CO_2_ accessibility. At the lowest L/B ratio, the higher mechanical strength is consistent with matrix characteristics that may have restricted CO_2_ transport, limiting access to the additional Ca-bearing phases introduced by CSA. Consequently, the greater availability of reactive calcium produced little additional progression of the carbonation front. At higher L/B ratios, greater CO_2_ accessibility may have allowed the additional Ca-bearing phases to participate more effectively in carbonation reactions, resulting in substantially higher CEF_x_ values. As noted above, this transport-related interpretation remains a proposed mechanism because the pore structure and CO_2_ transport properties were not directly measured.

Although carbonation depth was evaluated only for G0 and G20, CSA replacement level is also expected to influence the carbonation response through the availability of Ca-bearing phases. Increasing CSA content increases the amount of Ca-rich material potentially available for carbonate formation; however, this relationship should not necessarily be expected to be linear. Higher CSA replacement also reduces the amount of fly ash, accelerates early setting, alters fresh-state behavior, and may affect the transport characteristics of the hardened matrix. The carbonation response can therefore be considered to depend on the balance between Ca availability, influenced by CSA content, and CO_2_ accessibility, influenced by the L/B ratio and resulting matrix characteristics. Further measurements at intermediate CSA replacement levels would be required to establish the quantitative relationship between CSA content and *CEF_x_*.

### 3.5. X-Ray Diffraction Analysis (XRD)

**Figure 9:**
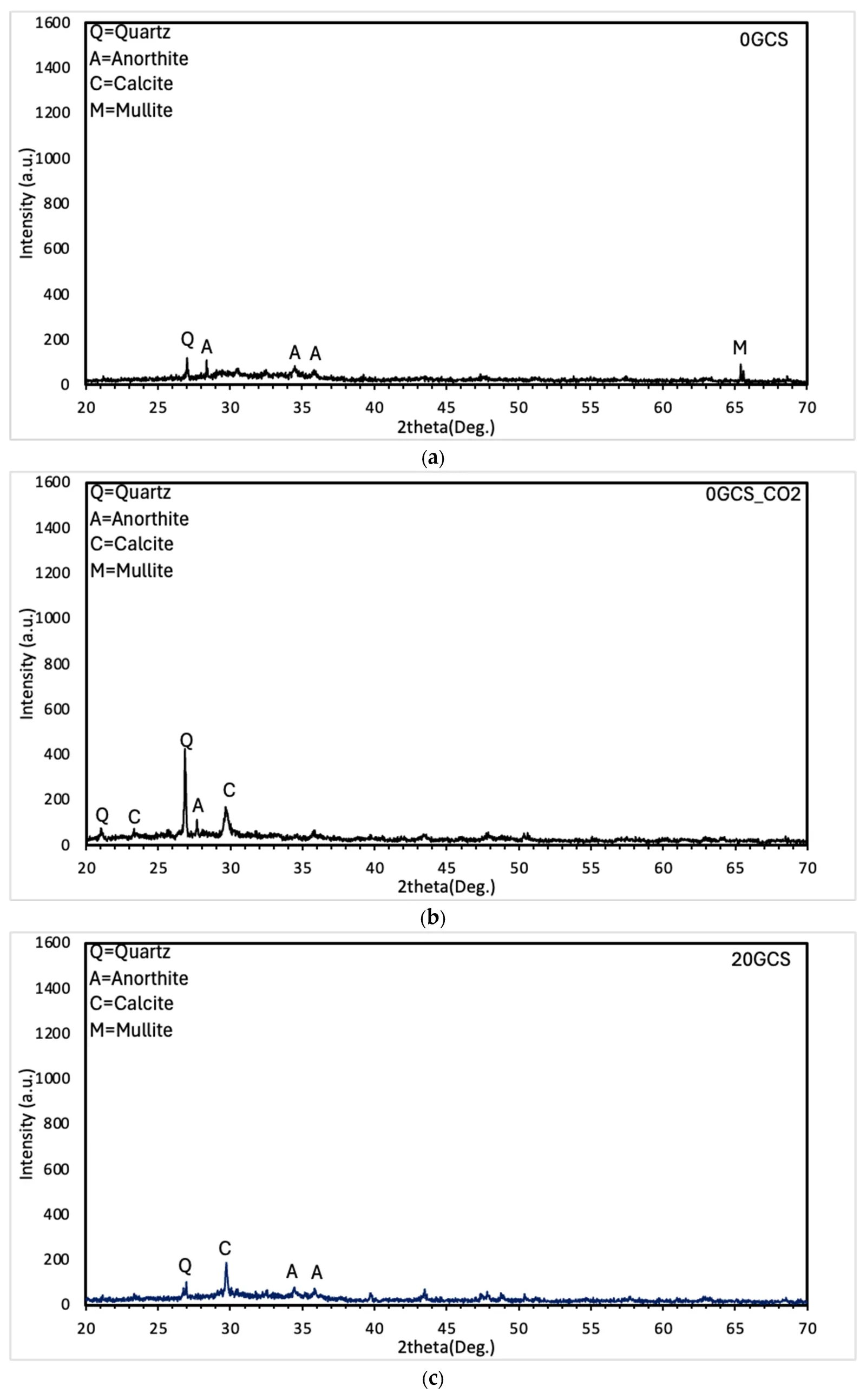

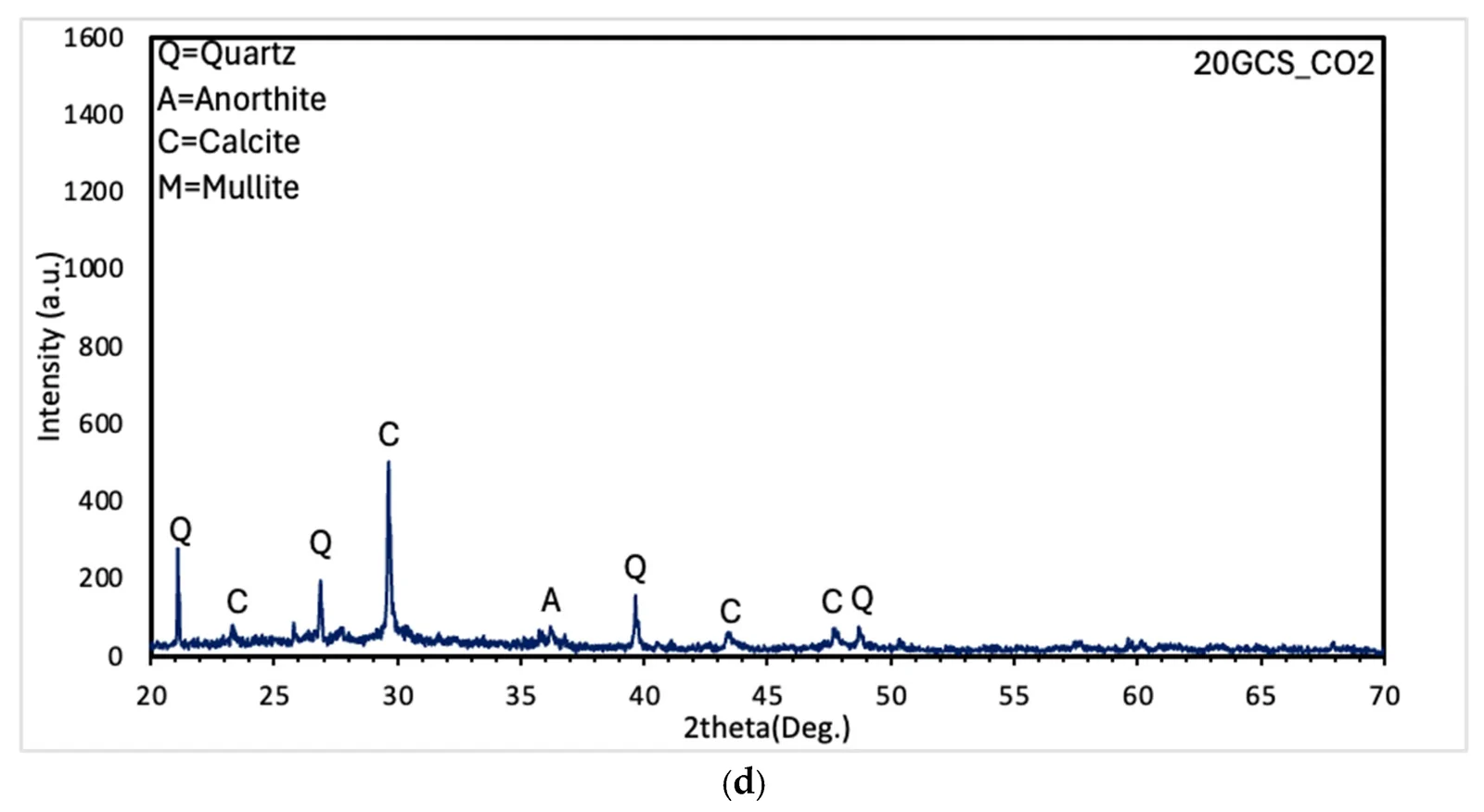
XRD patterns of alkali-activated mortars (L/B = 0.40): (**a**) G0 before accelerated carbonation, (**b**) G0 after accelerated carbonation, (**c**) G20 before accelerated carbonation, and (**d**) G20 after accelerated carbonation. (Q = quartz; A = anorthite; C = calcite; M = mullite).

Figure 9 presents the XRD patterns of the G0 and G20 alkali-activated mortars with an L/B ratio of 0.40 before and after accelerated carbonation. The principal crystalline phases identified in the specimens included quartz and calcite. Quartz reflections were predominantly observed near 2θ ≈ 26.6° and were retained in all specimens before and after carbonation, reflecting the presence of relatively inert crystalline constituents originating mainly from the fly ash and siliceous components of the mortar.

For the uncarbonated G0 mixture, the diffraction pattern was dominated by quartz reflections, whereas no prominent calcite peak was observed. Following accelerated carbonation, a calcite reflection appeared near 2θ ≈ 29.4°, indicating the presence of crystalline calcium carbonate following CO_2_ exposure. Because the XRD patterns were not normalized to a common reference intensity, the relative magnitude of this reflection cannot be used to quantify the amount of CaCO_3_ formed. Nevertheless, the appearance of the calcite-associated reflection after carbonation confirms a change in the crystalline carbonate assemblage of the G0 mortar.

The incorporation of 20% CSA resulted in a distinct carbonate-related diffraction response. Calcite was already detected in the uncarbonated G20 mixture, as indicated by the reflection near 2θ ≈ 29.4°. This initial calcite may have originated from residual CaCO_3_ that was not completely decomposed during calcination and/or from partial recarbonation of reactive Ca-bearing phases during cooling, storage, mixing, and curing. Accordingly, the calcite detected before accelerated exposure represents the initial carbonate content of the CSA-containing system and should not be attributed solely to carbonation occurring during chamber exposure.

Following accelerated carbonation, the G20 mixture exhibited clear changes in the calcite-associated diffraction features near 2θ ≈ 29.4°, together with additional calcite-related reflections. These changes are consistent with the formation of additional crystalline carbonate phases during accelerated CO_2_ exposure. However, because the diffraction patterns were not normalized to a common reference intensity, differences in absolute peak intensity between G0 and G20 are not interpreted quantitatively as differences in calcite abundance. Upon incorporation into the alkaline system, reactive Ca-bearing phases derived from CSA may undergo hydration and/or dissolution, increasing the availability of Ca^2+^ in the pore solution. During CO_2_ exposure, gaseous CO_2_ diffuses through the pore network and dissolves in the alkaline pore solution, where carbonate species are formed and subsequently react with available Ca^2+^, promoting the nucleation and precipitation of CaCO_3_.

Accordingly, CSA can be considered not merely as a partial replacement for fly ash, but as a Ca-rich reactive modifier that may provide additional Ca-bearing species available for mineral carbonation within the alkali-activated matrix. A plausible Ca-mediated carbonation pathway may involve hydration of CaO-containing phases, where present, followed by carbonation of the resulting calcium-bearing species:

CaO + H_2_O → Ca(OH)_2_ (2)

Ca(OH)_2_ + CO_2_ → CaCO_3_ + H_2_O (3)

In addition to this pathway, dissolved Ca^2+^ may react directly with carbonate species present in the pore solution, resulting in CaCO_3_ precipitation:

Ca^2+^ + CO_3_^2−^ → CaCO_3_(s) (4)

The pronounced increase in calcite reflections observed for G20 after accelerated carbonation is therefore consistent with the proposed Ca-mediated mineral carbonation pathway. The conversion of reactive Ca-bearing species into CaCO_3_ provides a potential mechanism for mineral CO_2_ fixation within the hardened alkali-activated matrix. Importantly, however, Equations (2)–(4) represent plausible reaction pathways rather than directly identified reaction sequences, as intermediate Ca-bearing species such as Ca(OH)_2_ were not independently quantified in the present study.

Overall, the XRD results provide qualitative evidence of changes in crystalline carbonate phases associated with accelerated CO_2_ exposure. The diffraction patterns were not normalized to a common reference intensity; therefore, differences in absolute peak intensity among specimens are not interpreted quantitatively as differences in phase abundance. Retrospective normalization to an arbitrary diffraction peak was not applied because the relative intensity of potential reference peaks may itself be affected by crystallinity, preferred orientation, sample preparation, and carbonation-induced changes. Accordingly, the XRD results are used primarily for crystalline-phase identification and qualitative assessment of carbonation-associated changes rather than as a direct quantitative measure of carbonate formation or CO_2_ uptake. The XRD findings are consequently interpreted together with the thermogravimetric results presented in Section 3.6, which provide complementary evidence of carbonation-associated changes in the CSA-containing system.

### 3.6. Thermogravimetric Analysis (TGA)

The TG/DTG results and corresponding mass-loss calculations were initially provided by an external testing laboratory and were subsequently verified using the raw TG data. The temperature intervals associated with the relevant thermal events differed among the specimens because the positions and widths of these events varied in the individual TG/DTG profiles. Recalculation of the raw TG data using the reported specimen-specific thermal intervals produced mass-loss values consistent with the laboratory results. The mass loss for each thermal interval was normalized to the initial sample mass at the beginning of the TG analysis and calculated as (*m_T_*_1_ − *m_T_*_2_)/*m*_0_ × 100, where *m_T_*_1_ and *m_T_*_2_ are the measured masses at the lower and upper temperature limits of the interval, respectively, and *m*_0_ is the initial sample mass. Therefore, the specimen-specific thermal intervals were retained rather than imposing a common fixed temperature range. Nevertheless, direct comparison of the resulting mass-loss values should be made cautiously because the temperature intervals differ among specimens and overlapping thermal processes may occur.

**Figure 10:**
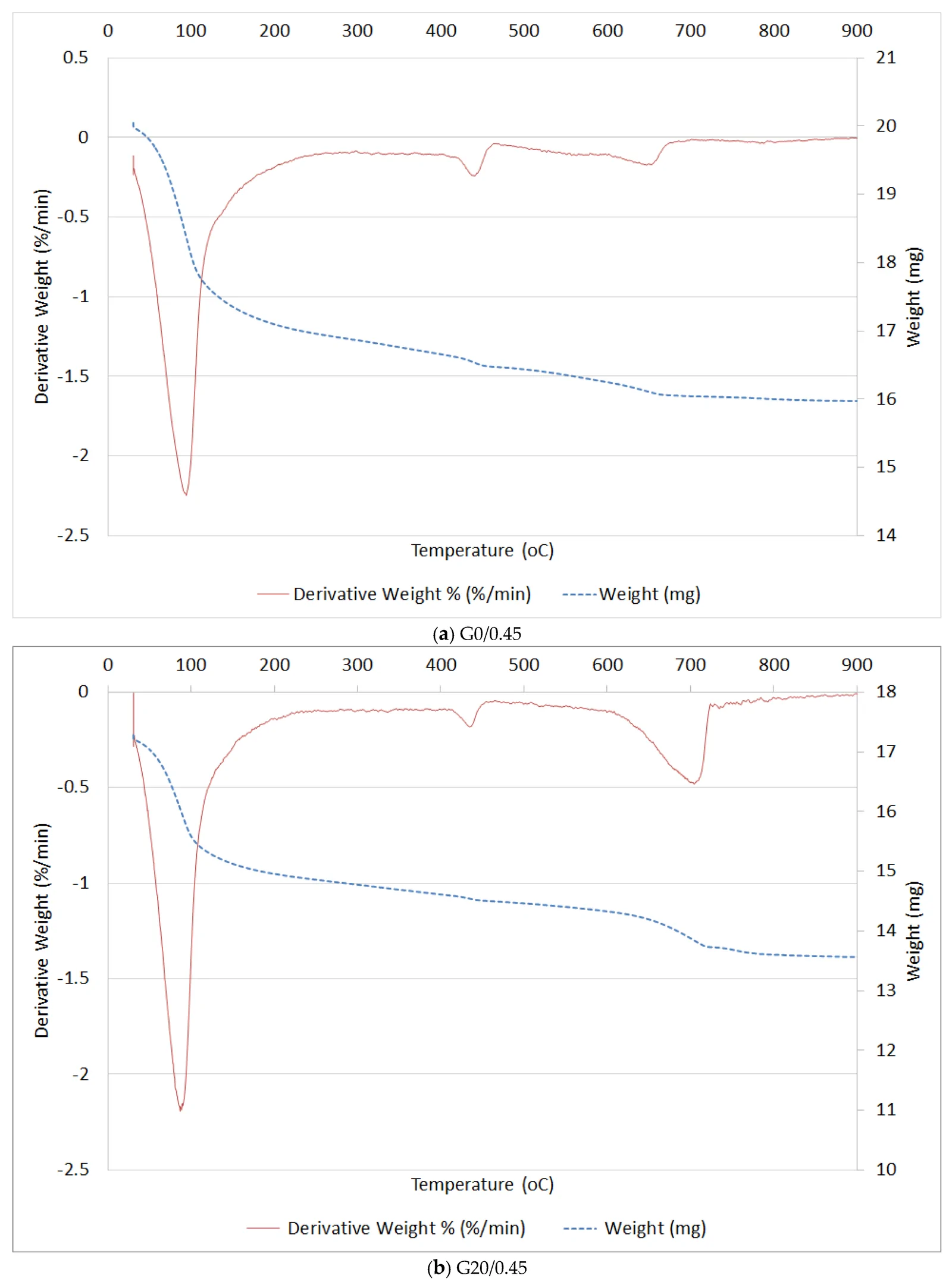

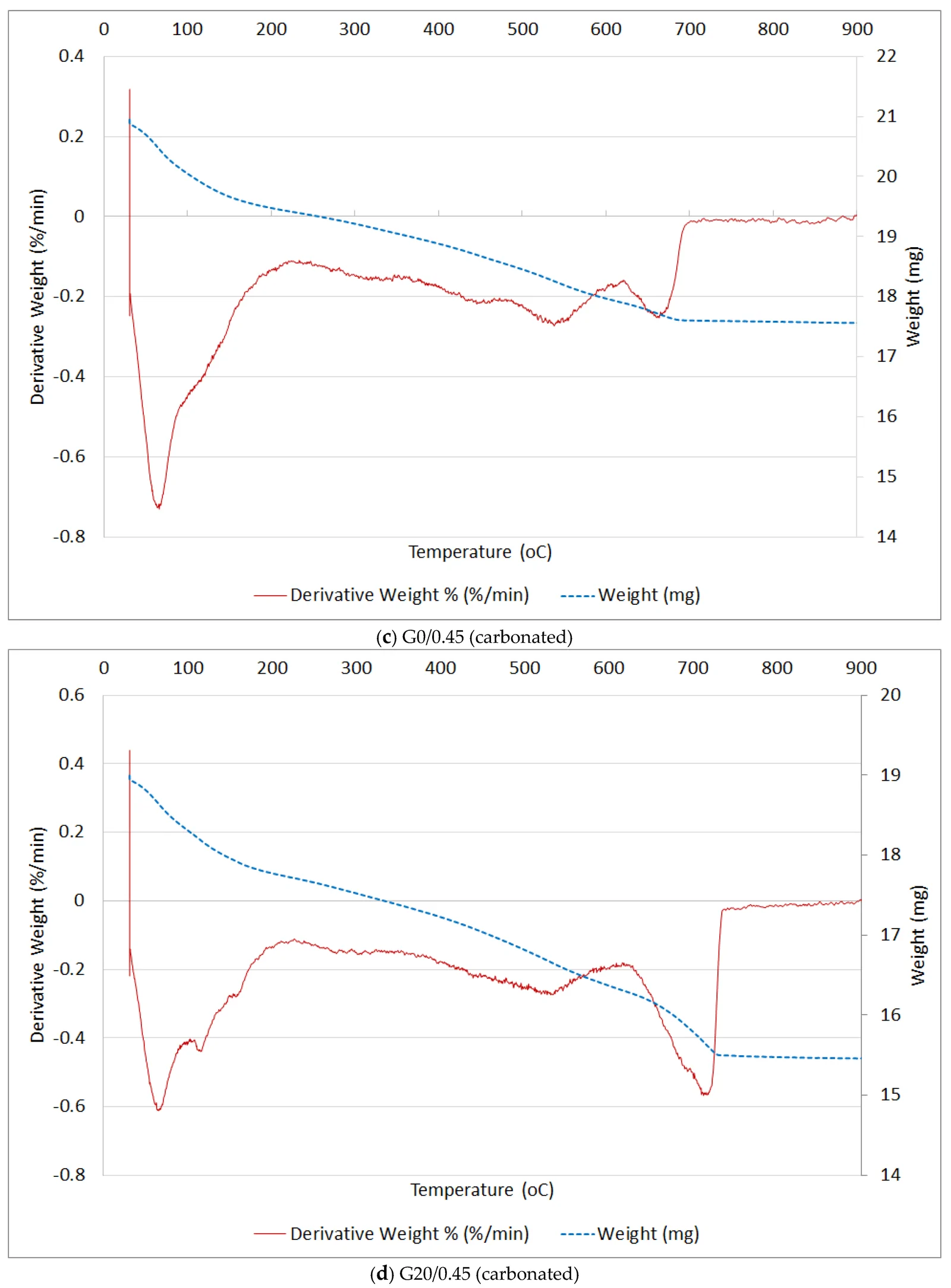
TG/DTG curves of (**a**) G0/0.45, (**b**) G20/0.45, (**c**) carbonated G0/0.45, and (**d**) carbonated G20/0.45.

Thermogravimetric analysis revealed clear differences in the thermal decomposition behavior of the AAB mixtures before and after accelerated carbonation (Figure 10 and Table 4). For the uncarbonated G0/0.45 mixture, the principal high-temperature decomposition region exhibited a mass loss of 1.954%. In comparison, G20/0.45 exhibited a considerably greater mass loss of 4.076%, indicating a greater initial contribution from thermally stable carbonate-bearing phases in the CSA-containing matrix.

Following accelerated carbonation, the high-temperature mass loss of G0/0.45 increased only slightly from 1.954% to 2.022%, corresponding to an increase of 0.068 percentage points, or approximately 3.5% relative to its initial value. In contrast, the corresponding mass loss of G20/0.45 increased from 4.076% to 4.981%, representing an increase of 0.905 percentage points, or approximately 22.2%. The considerably greater increase observed for G20/0.45 indicates that CSA incorporation produced a stronger carbonation-associated thermal response than that observed in the control AAB.

A consistent change was also observed in the intermediate-temperature region. The mass loss increased from 2.766% to 5.232% for G0/0.45 following carbonation and from 1.818% to 6.652% for G20/0.45, corresponding to increases of 2.466 and 4.834 percentage points, respectively. In addition to these quantitative changes, the DTG profiles of both carbonated mixtures differed markedly from those of the corresponding uncarbonated specimens, indicating that accelerated carbonation altered the distribution and thermal stability of the reaction products.

The greater carbonation-associated change in G20/0.45 is particularly notable when considered together with the carbonation-depth results. At L/B = 0.45, incorporation of 20% CSA increased the carbonation depth from 25.44 to 29.67 mm, corresponding to a CEF_x_ of 16.6%. In comparison, the increase in high-temperature mass loss following carbonation was 0.068 percentage points for G0/0.45 and 0.905 percentage points for G20/0.45. This contrast suggests that the influence of CSA was not limited to increasing the progression of the carbonation front. The additional Ca-bearing phases supplied by CSA also appear to have promoted a stronger mineralogical response within the carbonated material. Carbonation depth and TG/DTG therefore provide complementary information: the former indicates the progression of the carbonation front, whereas the latter reflects carbonation-associated changes in the thermal decomposition behavior of the matrix.

The stronger response of the CSA-containing mixture can be associated with its high calcium content, which provides additional Ca-bearing species capable of participating in carbonation reactions. This interpretation is consistent with the XRD results, which showed substantially increased calcite-related diffraction following accelerated carbonation. Taken together, the results support the proposed Ca-mediated mineral carbonation pathway in which reactive Ca-bearing phases supplied by CSA undergo hydration and/or dissolution, followed by reaction of available Ca species with dissolved carbonate species and precipitation of CaCO_3_, as described by Equations (1)–(3). Thus, the combined carbonation-depth, XRD, and TG/DTG results indicate that the mineral carbonation response is governed not only by CO_2_ penetration into the matrix but also by the availability of reactive Ca-bearing phases.

Nevertheless, the TG/DTG results should be interpreted cautiously. Mass loss in the intermediate-temperature region cannot be attributed exclusively to CO_2_ release because dehydration and dehydroxylation processes may overlap with decomposition of carbonate-bearing products. Similarly, differences in the high-temperature mass-loss regions are more appropriately interpreted as comparative evidence of carbonate-associated thermal changes rather than directly converted into absolute CO_2_ uptake. The presence of initial carbonate phases, overlapping thermal processes, and differences in the identified decomposition intervals prevent an unambiguous determination of captured CO_2_ without further quantitative phase analysis.

## 4. Conclusions

This study investigated the effects of calcined seashell ash (CSA) and liquid-to-binder (L/B) ratio on the fresh properties, mechanical performance, and mineral carbonation behavior of fly ash-based alkali-activated mortar. The results demonstrate that CSA can serve as a Ca-rich component that improves mechanical performance while enhancing the mineral carbonation response of the alkali-activated matrix.

Increasing the L/B ratio improved spread flow but prolonged initial setting and reduced mechanical strength. In contrast, increasing CSA content generally reduced spread flow and shortened initial setting time, with the accelerating effect becoming more evident at higher L/B ratios. CSA incorporation improved both compressive and flexural strengths up to a replacement level of 15%. G15 exhibited the highest mean strengths within each L/B group, with compressive-strength improvements of approximately 11.5–38.8% and flexural-strength improvements of approximately 102–107% relative to the corresponding G0 mixtures. Increasing the replacement level to 20% reduced the strength benefit, indicating that an appropriate balance between the Ca-rich CSA component and the aluminosilicate precursor is required.

Accelerated carbonation results further demonstrated that the influence of CSA depended strongly on the L/B ratio. At 20% CSA replacement, the carbonation-depth enhancement factor (CEF_x_) increased from only 1.2% at L/B = 0.35 to 13.2% and 16.6% at L/B = 0.40 and 0.45, respectively. This behavior indicates that the contribution of CSA to carbonation progression became more pronounced as the L/B ratio increased, suggesting an interaction between CO_2_ accessibility and the availability of Ca-bearing phases. However, because carbonation depth was measured only for G0 and G20, these results should not be interpreted as establishing a continuous CSA dose–response relationship. Moreover, carbonation depth should not be interpreted as a direct or proportional measure of chemically bound CO_2_.

XRD and TG/DTG analyses provided complementary evidence of enhanced mineral carbonation. Accelerated carbonation substantially increased calcite-related diffraction, particularly in the CSA-containing mixtures, while TG/DTG showed a stronger carbonation-associated thermal response for G20/0.45 than for the corresponding control. These findings indicate that CSA not only influenced the progression of the carbonation front but also provided additional Ca-bearing phases capable of participating in carbonate formation. Together with the carbonation-depth results, these mutually consistent observations support a Ca-mediated pathway in which CSA enhanced CO_2_ mineralization through increased carbonate formation under accelerated exposure conditions. Although these methods do not directly quantify the absolute amount of chemically bound CO_2_, they collectively demonstrate an enhanced mineral carbonation response rather than a quantitative CO_2_ sequestration capacity.

Overall, the results demonstrate the potential of calcined seashell waste as a functional Ca-rich component in fly ash-based alkali-activated materials, simultaneously contributing to mechanical performance and mineral carbonation. However, the present carbonation results were obtained under accelerated exposure, and the TG/DTG mass losses should not be interpreted as absolute CO_2_ uptake. The present study did not evaluate the net carbon balance associated with CSA production and use, including seashell calcination, grinding, and alkaline activator production; therefore, the observed enhancement in mineral carbonation should not be interpreted as demonstrating an overall reduction in embodied carbon. Further studies involving natural carbonation, intermediate CSA replacement levels, quantitative determination of CO_2_ uptake, and life-cycle carbon assessment are recommended to establish the long-term carbon-capture and environmental potential of the proposed system.

## Figures and Tables

**Table 1:** Chemical Composition of the Binder Materials.

Oxide	FA	Calcined Seashell Ash (CSA)
SiO_2_	31.85	4.93
Al_2_O_3_	15.89	3.92
Fe_2_O_3_	14.07	1.21
CaO	26.76	69.4
MgO	3.66	5.98
Na_2_O	1.95	9.61
K_2_O	0.96	0.06
SO_3_	2.45	1.22
P_2_O_5_	-	3.67
LOI	0.17	Not determined

Note: The CSA composition represents the average of two standard-less XRF measurements. The oxide-equivalent concentrations were normalized to 100% by the XRF quantification procedure. LOI of CSA was not independently determined. The reported CaO value represents total calcium expressed on an oxide-equivalent basis and should not be interpreted as reactive free CaO.

**Table 2:** Mix proportions of alkali-activated mortar (kg/m^3^).

Mix ID	FA	CSA	NaOH	Na_2_SiO_3_	River Sand	S/H	Actual W/B	Activator Na_2_O/B (%)
G0/0.35	772	–	135	135	1158	1	0.201	7.56
G10/0.35	695	77	135	135	1158	1	0.201	7.56
G15/0.35	656	116	135	135	1158	1	0.201	7.56
G20/0.35	618	154	135	135	1158	1	0.201	7.56
G0/0.40	772	–	154	154	1158	1	0.229	8.62
G10/0.40	695	77	154	154	1158	1	0.229	8.62
G15/0.40	656	116	154	154	1158	1	0.229	8.62
G20/0.40	618	154	154	154	1158	1	0.229	8.62
G0/0.45	772	–	174	174	1158	1	0.259	9.74
G10/0.45	695	77	174	174	1158	1	0.259	9.74
G15/0.45	656	116	174	174	1158	1	0.259	9.74
G20/0.45	618	154	174	174	1158	1	0.259	9.74

Note: G denotes ground CSA, while 0, 10, 15, and 20 indicate the percentage of CSA replacement by mass of total binder.

**Table 3:** Carbonation-depth enhancement factor of G20 relative to G0 after 28 days of accelerated carbonation.

L/B Ratio	x_G0_	x_G20_	Difference (mm)	CEF_x_ (%)
0.35	20.56	20.8	0.24	1.2
0.4	21.47	24.31	2.84	13.2
0.45	25.44	29.67	4.23	16.6

**Table 4:** Mass loss percentage at intermediate and high temperature regions.

Specimen	Condition	Intermediate Region (°C)	Mass Loss (%)	High-Temperature Region (°C)	Mass Loss (%)
G0/0.45	Before CB	270–545	2.766	545–890	1.954
G20/0.45	Before CB	320–530	1.818	530–730	4.076
G0/0.45/CB	After CB	340–595	5.232	595–886	2.022
G20/0.45/CB	After CB	245–590	6.652	590–889	4.981

## Data Availability

The original contributions presented in this study are included in the article. Further inquiries can be directed to the corresponding author.
